# The Effects of Heterologous Immunization with Prime-Boost COVID-19 Vaccination against SARS-CoV-2

**DOI:** 10.3390/vaccines9101163

**Published:** 2021-10-11

**Authors:** Tzu-Chuan Ho, Yi-Ming Arthur Chen, Hung-Pin Chan, Chin-Chuan Chang, Kuo-Pin Chuang, Che-Hsin Lee, Cheng-Hui Yuan, Yu-Chang Tyan, Ming-Hui Yang

**Affiliations:** 1 Department of Medical Imaging and Radiological Sciences, Kaohsiung Medical University, Kaohsiung 807, Taiwan; r090340@kmu.edu.tw; 2 Graduate Institute of Biomedical and Pharmaceutical Science, Fu Jen Catholic University, New Taipei City 242, Taiwan; 150110@mail.fju.edu.tw; 3 National Institute of Infectious Diseases and Vaccinology, National Health Research Institutes, Miaoli County 350, Taiwan; 4 Department of Nuclear Medicine, Kaohsiung Veterans General Hospital, Kaohsiung 813, Taiwan; hpchan@vghks.gov.tw; 5 Department of Nuclear Medicine, Kaohsiung Medical University Hospital, Kaohsiung 807, Taiwan; chinuan@kmu.edu.tw; 6 School of Medicine, Kaohsiung Medical University, Kaohsiung 807, Taiwan; 7 Neuroscience Research Center, Kaohsiung Medical University, Kaohsiung 807, Taiwan; 8 Department of Electrical Engineering, I-Shou University, Kaohsiung 840, Taiwan; 9 Graduate Institute of Animal Vaccine Technology, National Pingtung University of Science and Technology, Pingtung 900, Taiwan; kpchuang@g4e.npust.edu.tw; 10 Department of Biological Science, National Sun Yat-sen University, Kaohsiung 804, Taiwan; chlee@mail.nsysu.edu.tw; 11 Mass Spectrometry Laboratory, Department of Chemistry, National University of Singapore, Singapore 119077, Singapore; chmyuch@nus.edu.sg; 12 Graduate Institute of Medicine, College of Medicine, Kaohsiung Medical University, Kaohsiung 807, Taiwan; 13 Institute of Medical Science and Technology, National Sun Yat-sen University, Kaohsiung 804, Taiwan; 14 Department of Medical Research, Kaohsiung Medical University Hospital, Kaohsiung 807, Taiwan; 15 Center for Cancer Research, Kaohsiung Medical University, Kaohsiung 807, Taiwan; 16 Research Center for Environmental Medicine, Kaohsiung Medical University, Kaohsiung 807, Taiwan; 17 Department of Medical Education and Research, Kaohsiung Veterans General Hospital, Kaohsiung 813, Taiwan; 18 Center of General Education, Shu-Zen Junior College of Medicine and Management, Kaohsiung 821, Taiwan

**Keywords:** SARS-CoV-2, COVID-19, heterologous, vaccine safety, T-cell response

## Abstract

Coronavirus Disease 2019 (COVID-19) pandemic, which is caused by the severe acute respiratory syndrome coronavirus 2 (SARS-CoV-2), has become the global challenge. Reaching global herd immunity will help end the COVID-19 pandemic. However, vaccine shortage and vaccine hesitancy are the obstacles to achieve global herd immunity against SARS-CoV-2. The current homologous vaccine regimen is experimentally switching to heterologous vaccination at several study sites. However, the reactogenicity of heterologous ChAdOx1-S and mRNA vaccination against SARS-CoV-2 is still unclear. We have conducted a systematic review to summarize the current findings on the safety and immunogenicity of this heterologous vaccination and elucidate their implications against SARS-CoV-2. This systematic review was conducted by the guidelines of PRISMA. Articles were searched from PubMed and other sources (MedRixv and Google scholar) starting from 1 January to 5 September 2021. The search term was heterologous ChAdOx1-S and BNT162b2 or mRNA-1273 vaccination. Our review found that participants with ChAdOx1/BNT162b2, ChAdOx1-S/mRNA-1273 or BNT162b2/ChAdOx1-S did not have the serious adverse events seen with homologous vaccination. Participants with the heterologous regimen (ChAdOx1/BNT162b2, ChAdOx1-S/mRNA-1273 or BNT162b2/ChAdOx1-S), compared with those with two doses of ChAdOx1-S, have shown a more robust immune responses against SARS-CoV-2, such as higher levels of responsive antibodies or increased numbers of spike-specific T-cells. Nevertheless, these immune responses were slightly diminished in the recipients of BNT162b2/ChAdOx1-S. Also, the safety study of heterologous ChAdOx1-S/mRNA vaccination was based on small populations. Further studies to enclose diverse categories, such as race/ethnicity or geography, may be necessary. Overall, the heterologous immunization with ChAdOX1-S and the mRNA vaccine may improve the vaccine shortage related slow pace of reaching herd immunity, especially using the heterologous immunization with ChAdOx1-S/BNT162b2.

## 1. Introduction

Coronavirus Disease 2019 (COVID-19) pandemic, which is caused by the severe acute respiratory syndrome coronavirus 2 (SARS-CoV-2), has become the global challenge. The virus can infect host cells via the binding of cell receptor angiotensin-converting enzyme 2 (ACE2) with their spike proteins [1]. The common symptoms in COVID-19 patients include cough, fever, headache, fatigue, gastrointestinal discomfort, dyspnea, muscle pain, and olfactory/gustatory dysfunction [2]. The common medical images in asymptomatic COVID-19 patients included [^18^F]FDG-avid extrapulmonary lesions, such as multiple ground-glass opacities (GGOs), as well as [^18^F]FDG uptake in the mediastinal and hilar lymph nodes [3]. Since the COVID-19 outbreak in late December 2019, there are over 231.7 million cases and more than 4.7 million deaths in the entire world [4]. Several strategies such as city lockdowns, social distancing restrictions, or personal mask protection have been executed in many countries to control the spread of COVID-19 [5,6]. So far, the COVID-19 pandemic is still spreading globally. This seriously affected global health, society, and the economy [7]. The main treatment options for COVID-19 are antiviral (against virus replication) and immunomodulatory/anti-inflammatory agents (to avoid tissue damage). These are mostly symptomatic treatments, which include the usage of dexamethasone, tocilizumab, remdesivir or chloroquine/hydroxychloroquine, but none of these is ideal [8,9]. Thus, the prevention (vaccination) becomes valuable. Full immunization with vaccines is considered as a critical strategy against SARS-CoV-2 [10]. It can help end the COVID-19 pandemic by achieving global herd immunity. Thus, vaccine development has been initiated through various platforms in 2020.

At end of July 2021, according to information from the WHO website and clinical results [11,12,13,14,15,16,17,18,19,20,21,22,23,24], multiple vaccines can provide protection for COVID-19 as shown in Table 1. The types of these vaccines include two recombinant adenovirus vaccines (ChAdOx1-S and Ad26.COV2-S), one heterologous recombinant adenovirus vaccine (Gam-COVID-Vac), two mRNA vaccines (BNT162b2 and mRNA-1273), two inactivated virus vaccines (BBIBP-CorV and CoronaVac), and one recombinant protein vaccine (NVX-CoV2373). According to Table 1, for full immunization with most vaccines (excluded from Ad26.COV2-S and Gam-COVID-VaC), people need to be inoculated with two doses of the same vaccines (i.e., homologous vaccination, homologous vaccine regimen, or homologous prime-boost schedules) with an interval of 14 days to three months. As of 26 September 2021, only 32.86% of the world was fully immunized with the COVID-19 vaccine [25]. Vaccine shortage delays the schedule for the second dose of homologous vaccination and postpones the achievement of global herd immunity.

Since the vaccination is voluntary, the rate of full immunization also depends on population acceptance [26]. Accompanied with the increased numbers of vaccinated people, more uncommon serious adverse events are found in the immunization with ChAdOx1-S, Ad26.COV2-S, BNT162b2, mRNA-1273, or NVX-CoV2373 (Table 1). As of the beginning of March 2021, the life-threatening cerebral or other venous sinus thrombosis related to homologous ChAdOx1-S vaccination (ChAdOx1-S/ChAdOx1-S) have frequently occurred, especially in young women [27,28,29]. Those uncommon serious adverse events may trigger the decline of COVID-19 vaccination, including those people who had received the first dose of ChAdOx1-S. These events have enhance the forming of vaccine hesitancy, which is a behavior of a delayed acceptance or refusal of vaccination despite the vaccination services which are available [26,30]. Subsequently, vaccine hesitancy may become one of the key factors to delay the completion of global herd immunity.

The status of vaccine shortage and vaccine hesitancy can affect the timing of global herd immunity. The use of heterologous vaccines may be beneficial for earlier reduction of the COVID-19 pandemic. Since the vaccine shortage and vaccine hesitancy slow the rate of herd immunity, the current vaccine regimen has been switched to heterologous vaccination from homologous vaccination [31,32]. Several studies have been reported for the safety and immunogenicity of heterologous ChAdOx1-S and mRNA vaccination (heterologous combing with first dose/second dose of ChAdOx1-S/mRNA and mRNA vaccine/ChAdOx1-S) [33,34,35,36,37,38,39,40,41]. However, the implication of heterologous ChAdOx1-S and mRNA vaccination against COVID-19 is still unclear. The safety and immunogenicity for each vaccine regmien are crtical factors to combat COVID-19. Hence, we conduct a systematic review to summarize the current findings on the safety and immuno-genicity of this heterologous vaccination and elucidate their implications against COVID-19.

Our systematic review showed that populations with ChAdOx1/BNT162b2, ChAdOx1-S/mRNA-1273 or BNT162b2/ChAdOx1-S did not have serious adverse events. Comparison of two doses of ChAdOx1-S vaccination, the participants with ChAdOx1/BNT162b2, ChAdOx1-S/mRNA-1273 or BNT162b2/ChAdOx1-S have shown a robust immune response against SARS-CoV-2, such as a high level of Spike-specific IgG titer, a high neutralization antibody titer, or a strong Spike-specific T-cell response. In addition, the immune response in the population with ChAdOx1-S/BNT162b2 was better than the population with BNT162b2/ChAdOx1-S. In order to analyze the evolution of heterologous ChAdOx1-S and mRNA vaccination, the present study aimed at assessing knowledge about the heterologous vaccination among studies from Europe (Sweden, UK and Germany) with the focal points of safety and immunogenicity.

## 2. Article Screening

**Figure 1:**
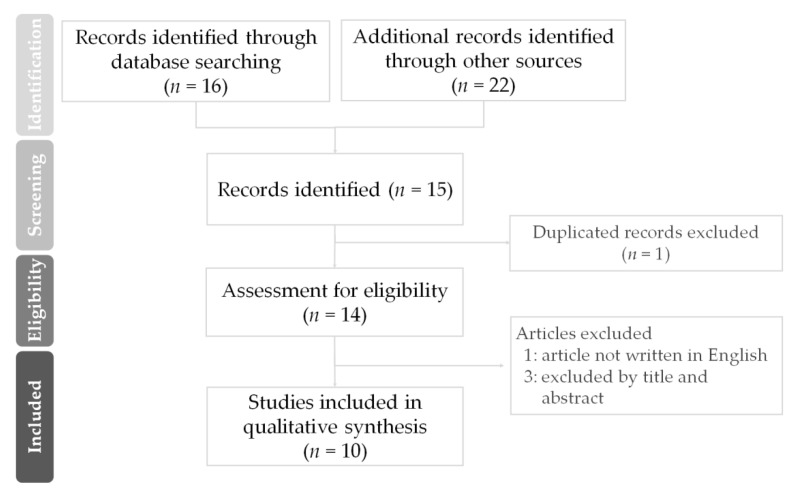
The flow diagram of PRISMA showed the processing of article screening in this study.

This systematic review was conducted by the guidelines of PRISMA (Figure 1). Articles were searched from PubMed and other sources (MedRixv and Google scholar) starting from from 1 January to 5 September 2021. The search term was heterologous ChAdOx1-S and BNT162b2 or mRNA-1273 vaccination. This review was absorbed in the studies regarding the safety and immunogenicity of heterologous vaccination. Duplicated articles were removed. All authors reviewed the articles and excluded irrelevant articles by the title and abstracts. The language of all articles was restricted to English. Our systematic review finally included ten articles (two short comments [33,34], six clinical studies [35,36,37,38,39,40], one observation study [41] and one prospective study [42]) from fourteen potentially relevant citations. The two brief reports depict the design and findings in two clinical studies [36,37], which were included in this review.

## 3. Safety and Immunogenicity

Current studies of heterologous ChAdOx1-S and mRNA vaccination are shown in Table 2, including five clinical studies [35,36,37,38,39,40], one observation study [41] and one prospective study [42]. Those studies were individually processed in Sweden, UK, Spain, and Germany. The interventions for heterologous vaccination of ChAdOx1-S and mRNA vaccine are combined as two doses. There are four types of heterologous groups according to the order of dose inoculated in these studies (1st dose/2nd dose), including ChAdOx1-S/BNT162b2 [35,36,37,38,39,40,41,42], BNT162b2/ChAdOx1-S [35,39], ChAdOx1-S/mRNA-1273 [34], and ChAdOx1-S/BNT162b2 or mRNA-1273 [40,42] (Table 2).

The safety of heterologous ChAdOx1-S and mRNA vaccination was reported in five clinical studies [35,36,37,38,39,40] and one prospective study [42]. Two clinical studies had separately enrolled the participants for heterologous ChAdOx1-S/BNT162b2 vaccination from Spain (*n* = 451) [37] and Germany (*n* = 26) [38]. Two clinical studies utilized the same participants from the UK to separately evaluate the vaccine safety within seven and 28 days after the boost (*n* = 110) [34,39]. Another one was estimated the vaccine effectiveness (VE) of heterologous vaccine (ChAdOx1 with mRNA vaccine as the second dose) from Denmark [40]. The interval for heterologous vaccination of ChAdOx1-S/BNT162b2 was 8–12 weeks for the study in Spain [37], eight weeks for that in Germany [38], four weeks for that in the United Kingdom [35,39], and 82 days in Denmark [40]. For the prospective study, the individuals were screened who received the ChAdOx1-S/BNT162b2 with the 10–12-week vaccine interval (*n* = 104). Although the intervals were dissimilar, these studies all reported no serious adverse events regarding heterologous ChAdOx1-S/BNT162b2 vaccination after one [42], seven [36,37], 28 days [39], or more than one day [38].

The two clinical studies from the United Kingdom also enrolled the participants for heterologous BNT162b2/ChAdOx1-S vaccination with the four-week interval. All participants with heterologous BNT162b2/ChAdOx1-S vaccination did not present vaccine-related serious adverse events within seven [36] and 28 days [39] after boost (*n* = 114). The occurrence of serious adverse events was not related to the vaccination order of BNT162b2 and ChAdOx1-S.

A clinical study in Sweden further reported no serious adverse events in the participants with the heterologous ChAdOx1-S/mRNA-1273 vaccination on day 7 to day 10 after the boost [36]. This was also found in the individuals with heterologous ChAdOx1-S/mRNA-1273 or ChAdOx1-S/BNT162b2 vaccination within seven days after the boost (*n* = 96). Regardless of interventions or intervals of heterologous ChAdOx1-S and mRNA vaccination, there were no serious adverse events regarding this heterologous vaccine regimen. However, the serious adverse events are still listed in the safety concerns of ChAdOx1-S and mRNA vaccine as very rare, which have been only observed in one per 100,000 to 250,000 ChAdOx1-S vaccinated people [43] and 2.5 to 24 per 10,000,000 mRNA vaccinated people [15,16,17]. Current studies in the safety of heterologous ChAdOx1-S and mRNA vaccination were based only on small populations. More clinical studies are needed to evaluate the safety of heterologous vaccination.

The immunogenicity of heterologous ChAdOx1-S and mRNA vaccination is important for COVID-19 protection. Current studies have evaluated the immunogenicity of heterologous ChAdOx1-S and mRNA vaccination via detecting the level of SARS-CoV-2-specific IgG, the ability of neutralization antibody against wild type or variant SARS-CoV-2 or Spike-specific T-cell immune response (Table 2). These five studies reported the levels of SARS-CoV-2-specific IgG between homologous and heterologous vaccine groups [35,38,39,40,41,42]. Four of those studies independently showed that the level of SARS-CoV-2-Spike-specific IgG was significantly higher (in people who received ChAdOx1-S, then the boost of BNT162b2 or mRNA-1273) than that in people having homologous ChAdOx1-S/ChAdOx1-S vaccination regardless of the inoculating intervals [35,39,40,41,42,43]. Moreover, this IgG level of the heterologous vaccination groups was similar to or higher than that of the homologous vaccination with BNT162b2/BNT162b2 [38,39,42] or mRNA 1273/mRNA 1273 [41]. A similar observation was found on the level of SARS-CoV-2- receptor-binding domain-specific IgG [35,42]. One clinical study further showed that the heterologous ChAdOx1-S/BNT162b2 vaccination could induce a higher level of SARS-CoV-2-Spike-specific IgG in comparison to the heterologous BNT162b2/ChAdOx1-S vaccination [39].

Four studies reviewed as following, have shown the efficacy of neutralization antibody against wild type SARS-CoV-2 between homologous and heterologous vaccine groups [35,38,39,41]. The 50% of pseudovirus neutralization titer (PVNT_50_) in the heterologous ChAdOx1-S/BNT162b2 vaccinated people was significantly higher than that in the homologous ChAdOx1-S/ChAdOx1-S vaccinated people and was equal or similar to that in the homologous BNT162b2/BNT162b2 vaccinated people regardless of the inoculating intervals [38,39]. One clinical study showed that the percentage of inhibition of surrogate virus neutralization antibody in the groups of heterologous ChAdOx1-S/BNT162b2 or mRNA1273 vaccination was significantly higher than that in the homologous ChAdOx1-S/ChAdOx1-S vaccination [41]. Another study that utilized the real virus neutralization test found similar results between the groups of the heterologous ChAdOx1-S/mRNA1273 vaccination and the homologous ChAdOx1-S/ChAdOx1-S vaccination [35]. In addition, the efficacy of neutralization antibody against wild type SARS-CoV-2 in the heterologous ChAdOx1-S/BNT162b2 vaccination group was better than that of the heterologous BNT162b2/ChAdOx1-S vaccination group [39].

The efficacy of neutralization antibody against variant SARS-CoV-2 between homologous and heterologous vaccine groups which was detected in three studies [35,38,42]. The heterologous vaccination of ChAdOx1-S/BNT162b2 or ChAdOx1-S/mRNA-1273 produced a better neutralization capacity against alpha- or beta- SARS-CoV-2 when compared with that of the homologous vaccination of ChAdOx1-S/ChAdOx1-S or BNT162b2/BNT162b2 [35,38,42].

There were three studies which showed the Spike-specific T-cell immune response between the heterologous vaccine group and the homologous vaccine group [39,41,42]. This was detected by the production of IFN-γ or the level of IFN-γ+ T-cell in PBMC after Spike stimulation. The production of IFN-γ was significantly higher in the heterologous ChAdOx1-S/BNT162b2 vaccination than that in the homologous ChAdOx1-S/ChAdOx1-S [42]. Although the level of IFN-γ+ T-cell in PBMC was not significantly higher in the heterologous ChAdOx1-S/BNT162b2 group [39], the other study showed that the level of antigen-specific T-cells (CD69+ IFN-γ+ CD8+ T-cell) was significantly higher in the heterologous ChAdOx1-S/BNT162b2 or mRNA-1273 group [43]. Overall, the heterologous ChAdOx1-S and mRNA vaccination could induce a robust immune response against COVID-19 in comparison with the homologous ChAdOx1-S/ChAdOx1-S.

## 4. Discussion

This systematic review aimed to summarize the current findings on the safety and immunogenicity of this heterologous vaccination to elucidate their implications against COVID-19. The line of our systematic review showed that the heterologous combination with ChAdOx1-S and mRNA vaccine can induce a robust immune response to eliminate the SARS-CoV-2. It is similar to a robust humoral and cellular response induced by the heterologous vaccination of Gam-COVID-Vac [23,24]. It indicates the heterologous ChAdOx1-S and mRNA vaccination can enhance the immune response against SARS-CoV-2. In addition, the immune response in the population with ChAdOx1-S/BNT162b2 was better than the population with BNT162b2/ChAdOx1-S. Our systematic review cannot demonstrate the efficacy of heterologous ChAdOx1-S and mRNA vaccination; however, people with heterologous vaccination of Gam-COVID-Vac present 91.6% efficacy against COVID-19 [23,24]. The level of neutralization antibody has been reported to correlate with the clinical protection [44]. It can be expected that people with heterologous ChAdOx1-S and mRNA vaccination have a good protective effect for COVID-19. Current studies in the safety of heterologous ChAdOx1-S and mRNA vaccination were based on small populations. The incidence of serious cases in people who received ChAdOx1-S or mRNA vaccine were very rare [39]. Despite of no serious adverse events in the people with heterologous ChAdOx1-S and mRNA vaccination, it cannot really reflect the incidence of serious cases in the real world. Overall, the heterologous ChAdOx1-S and mRNA vaccination is an excellent strategy of vaccination to control the COVID-19 pandemic, but it is also accompanied by a potential safety concern.

The immune response in the population with ChAdOx1-S/BNT162b2 was better than the population with BNT162b2/ChAdOx1-S. This finding indicated that a strong immune response can be induced in the people who had received the first dose of ChAdOx1-S with a BNT162b2 boost. Although the mechanisms are unknown, it provides the priority order of heterologous ChAdOx1-S and BNT162b2. It is helpful for vaccine management in the countries who are starting to implement heterologous ChAdOx1-S and BNT162b2 vaccination. Because there are studies regarding the safety and immunogenicity of heterologous mRNA-1273/ChAdOx1-S vaccination, we do not know the ability of this regime to eliminate the SARS-CoV-2 and control COVID-19 pandemic. Additionally, the heterologous combination in this review is restricted to the ChAdOx1-S and mRNA vaccine, in which the implications of heterologous combination with other vaccines are not addressed.

## 5. Conclusions

Reaching global herd immunity will help stop the spread of COVID-19, but the vaccine shortage and vaccine hesitancy are the obstacles to achieve such immunity against SARS-CoV-2. This review suggested that the heterologous ChAdOx1-S and BNT162b2 or mRNA-1273 vaccination is a feasible and practical approach to end the COVID-19 pandemic. Although stronger immune responses can be induced without having serious adverse events, an extensive follow-up study may be needed to verify vaccine-induced protection against COVID-19 and related hospitalization/death.

## Figures and Tables

**Table 1:** List of valid vaccines for COVID-19.

Vaccine Name	Developer Country	Manufacturer	Vaccine Type	Storage Temperature/Shelf Life	Number of Doses	Interval between Doses	Vaccine Efficacy/Age (y)	Serious Adverse Event	Reference
**ChAdOx1-S (AZD1222)**	UK	AstraZeneca, SK Bioscience, and Serum Institute of India	Recombinant adenovirus vector	2–8 °C/6 months	2	Day 28–84	63%/≥18	Cerebral venous sinus thrombosis (CSVT) and other venous thrombosis with thrombocytopenia syndrome	[11,12,13,14]
**Ad26.COV2-S**	USA and Europe	Janssen Pharmaceutical and Johnson & Johnson	Recombinant adenovirus vector	2–8 °C/4.5 months −20 °C/2 years	1	Day 0	66.9%/≥18	Cerebral venous sinus thrombosis (CSVT) and other venous vthrombosis with thrombocytopenia syndrome	[12,13,14,15]
**BNT162b2**	USA and Germany	Pfizer and BioNTech	mRNA	−70 °C/6 months	2	Day 21	92%/≥16	Anaphylaxis and myocarditis	[12,14,16,17]
**mRNA-1273**	USA and Europe	Moderna Biotech	mRNA	2–8 °C/1 month −20 °C/6 months	2	Day 28	94.1%/≥18	Myocarditis, anaphylaxis, and other serious allergic reactions	[12,14,17,18,19]
**BBIBP-CorV (BIBP vaccine or Sinopharm COVID-19 vaccine)**	China	Beijing Institute of Biological Products and Sinopharm	Inactivated virus	2–8 °C/2 years	2	Day 14	78.1%/18–59	No adverse reports **	[12,14]
**CoronaVac**	China	Sinovac	Inactivated virus	2–8 °C/2 years	2	Day 14	50.7%/18–59 51.1%/≥60	No adverse reports **	[12,14,20,21]
**NVX-CoV2373**	USA	Novavax	Recombinant protein	2–8 °C/not reported	2	Day 21	89.7%/≥18 *	Myocarditis	[22]
**Gam-COVID-Vac (Sputnik V)**	Russia	Gamaleya Research Institute of Epidemiology and Microbiology	Heterologous recombinant adenovirus vector	2–8 °C for dry form or −18.5 °C for liquid form/not reported	1st dose rAd5; 2nd dose rAd6	Day 21	91.6%/≥18	No adverse reports **	[23,24]

* The vaccine efficacy was based on combination of vaccination with one or two dose. ** Accorded to WHO website.

**Table 2:** Studies of heterologous ChAdOx1-S with mRNA vaccination.

Reference	Country	Design	Interval between Doses	Intervention (1st/2nd Dose)	Results
**Johan N. et al., 2021** [35]	Sweden	An open, multicenter phase IV study	9–12 weeks	Homologous vaccine group: ChAdOx1-S/ChAdOx1-S (*n* = 37, 28- to 62-year-old) Heterologous vaccine group: ChAdOx1-S/mRNA-1273 (*n* = 51, 23- to 59-year-old)	S-specific and RBD-specific IgG geometric mean titers At the day of the 2nd dose inoculation, the similar titer of S-specific and RBD-specific IgG between two groups At D7 to D10 after 2nd dose inoculation, S-specific and RBD-specific IgG titers in the ChAdOx1-S/mRNA-1273 were separately 115-fold and 125-fold of that on the day of the 2nd dose inoculation, and that was 5-fold in the ChAdOx1-S/ChAdOx1-S At D30 after 2nd dose inoculation, S-specific and RBD-specific IgG titers in two groups were the same with that on D7 to D10 time point. Neutralization antibody against wild type SARS-CoV-2 At the day of 2nd dose inoculation, the titer of ID_50_ was similar between two groups At D7 to D10 after 2nd dose inoculation, the titer of ID_50_ in the ChAdOx1-S/mRNA-1273 was 20-fold of that on the day of 2nd dose inoculation and it was 2-fold in the ChAdOx1-S/ChAdOx1-S At D30 after 2nd dose inoculation, the titer of ID_50_ in two groups was 1.6 to 1.7-fold of that on D7 to D10 time point, but it was not significant Neutralization antibody against B.1.351, Beta variant SARS-CoV-2 At the D7 to D10 after 2nd dose inoculation, the ChAdOx1-S/mRNA-1273 had induced the antibodies that could neutralize the B.1.351, Beta variant SARS-CoV-2, but the ChAdOx1-S/ChAdOx1-S could not induce potent antibodies against this variant Adverse events (on the D7 to D10 after 2nd dose inoculation) No serious adverse events were reported in two groups The incidence of systemic adverse events such as fever, headache, chills and injection site pain, was frequently found in the ChAdOx1-S/mRNA-1273 than that in the ChAdOx1-S/ChAdOx1-S The grade of adverse events was not statistically significant different between two groups
**Robert, H.S. et al., 2021** [33,34,36]	UK	A single-blind, randomized, multicenter phase II study	4 weeks	Homologous vaccine group (50- to 69-year-old): ChAdOx1-S/ChAdOx1-S (*n* = 115); BNT162b2/BNT 162b2 (*n* = 110) Heterologous vaccine group (50- to 69-year-old): ChAdOx1-S/BNT162b2 (n = 110); BNT162b2/ChAdOx1-S (*n* = 114)	Adverse events No serious adverse events reported in all groups within 7 days after inoculation The systemic adverse events were more frequently found in the heterologous vaccine groups than that in their homologous vaccine groups within 2 days after inoculation
**Alberto, M.B. et al., 2021** [34,37]	Spain	An open-label, randomized, controlled multicenter phase II study	8–12 weeks	Without homologous vaccine group, only 1 dose of ChAdOx1-S (*n* = 226, 18- to 60-year-old): Heterologous vaccine group (18- to 60-year-old): ChAdOx1-S/BNT162b2 (*n* = 451)	S-specific and RBD-specific IgG geometric mean titers At the day of 2nd dose inoculation, the similar titer of S-specific and RBD-specific IgG between two groups The titer of S-specific and RBD-specific IgG in the 1st dose of ChAdOx1-S on the day of 2nd dose inoculation, which was similar to that on D7 and D14 after inoculation At D7 and D14 after 2nd dose inoculation, both S-specific and RBD-specific IgG titers in the ChAdOx1-S/BNT162b2 were significantly higher than that in the 1st dose of ChAdOx1-S Neutralization antibody against pseudovirus-SARS-CoV-2 At the day of 2nd dose inoculation, PVNT50 was similar between two groups At D14 after 2nd dose inoculation, PVNT50 in the ChAdOx1-S/BNT162b2 were 45-fold of that in the 1st dose of ChAdOx1-S S-specific T cell immune response At the day of 2nd dose inoculation, production of IFN-γ was similar between two groups At the D14 after 2nd dose inoculation, production of IFN-γ in the ChAdOx1-S/BNT162b2 was significantly higher than in the 1st dose of ChAdOx1-S Adverse events No serious adverse events were reported in heterologous vaccine group Incidence of systematic adverse events were more than others in ChAdOx1-S/BNT162b2 within D7 after 2nd dose of inoculation There was no data regarding the difference in the incidence of adverse events between the two group
**Tina S. et al., 2021** [41]	Germany	Observation study	9–12 weeks: ChAdOx1-S/ChAdOx1-S; ChAdOx1-S/BNT162b2 or mRNA-1273 3–6 weeks: BNT162b2/BNT162b2 or mRNA-1273/mRNA-1273	Homologous vaccine group: ChAdOx1-S/ChAdOx1-S (*n* = 55, 36- to 61-year-old); BNT162b2/BNT162b2 or mRNA-1273/mRNA-1273 (*n* = 62, 29- to 52-year-old) Heterologous vaccine group: ChAdOx1-S/BNT162b2 or mRNA-1273 (*n* = 96, 30- to 59-year-old)	S-specific IgG geometric mean titers: At the D14 after 2nd dose inoculation, the titer of S-specific IgG was similar between ChAdOx1-S/BNT162b2 or mRNA-1273 and 2 dose of BNT162b2 or mRNA-1273, that was significantly higher than that in the ChAdOx1-S/ChAdOx1-S Neutralization antibody against SARS-CoV-2 by surrogate virus neutralization test At the D14 after 2nd dose inoculation, the percentage inhibition of neutralization antibody was similar between ChAdOx1-S/BNT162b2 or mRNA-1273 and 2nd dose of BNT162b2 or mRNA-1273, that was significantly higher than that in the ChAdOx1-S/ChAdOx1-S S-specific T cell immune response: At the D14 after 2nd dose inoculation, percentage of CD69+ IFN-γ+ CD4+ T cells was similar between ChAdOx1-S/BNT162b2 or mRNA-1273 and 2nd dose of BNT162b2 or mRNA-1273, that was significantly higher than that in the ChAdOx1-S/ChAdOx1-S The percentage of CD69+ IFN-γ+ CD8+ T cells was significantly higher than that in both ChAdOx1-S/ChAdOx1-S and 2nd dose of BNT162b2 or mRNA-1273 Adverse events (within D7 after 2nd inoculation): No serious adverse events were reported in heterologous vaccine group The incidence of adverse events in the ChAdOx1-S/BNT162b2 or mRNA-1273 was similar to that in the 2 doses of BNT162b2 or mRNA-1273, but more than that in the 2 doses of ChAdOx1-S The incidence of adverse events in the ChAdOx1-S/BNT162b2 was similar to that in the ChAdOx1-S prime
**Rüdiger G. et al., 2021** [38]	Germany	Clinical study	8 weeks	Homologous vaccine group: BNT162b2/BNT162b2 (*n* = NR, 25-to 55-year-old) Heterologous vaccine group: ChAdOx1-S/BNT162-b2 (*n* = 26, 25- to 46- year-old)	S-specific IgG titer: At the D14–19 after 2nd dose inoculation, this titer in the ChAdOx1-S/BNT162b2 were separately significantly higher than that at the day of 2nd dose inoculation and that in the 2 doses of BNT162b2 at D13–15 after 2nd dose inoculation Neutralization antibody against pseudovirus-wild type-SARS-CoV-2 At the D14–19 after 2nd dose inoculation, the PVNT50 in the ChAdOx1-S/BNT162b2 were separately significantly higher than that at the day of 2nd dose inoculation and that in the 2 doses of BNT162b2 at D13–15 after 2nd dose inoculation Neutralization antibody against pseudovirus-variant-SARS-CoV-2 PVNT50 against alpha- and beta-SARS-CoV-2 in in the ChAdOx1-S/BNT162b2 at D14–19 after 2nd dose inoculation, was separately higher than that in the 2 doses of BNT162b2 at D13–15 after 2nd dose inoculation, but the PVNT50 against delta-SARS-CoV was similar between two groups S-specific T cell immune response: A significantly high percentage of S-specific IFN-γ+CD4 or CD8 T cells the ChAdOx1-S/BNT162b2 at the D6–11 and D14–19 after 2nd dose inoculation in comparison to that at D2 before 1st dose inoculation There was no data regarding the difference on S-specific T cell immune response between two groups Adverse events (lasting than D1 after boost): No serious adverse events were reported in heterologous vaccine group There is no data regarding the difference in the incidence of adverse events between the two groups
**Xin Xue L. et. al., 2021** [39]	UK	A single blinded, randomized, multicenter, phase II, non-inferiority study	4 weeks	Homologous vaccine group (50- to 69-year-old): ChAdOx1-S/ChAdOx1-S (*n* = 112); BNT162b2/BNT162b2 (*n* = 110) Heterologous vaccine group (50- to 69-year-old): ChAdOx1-S/BNT162b2 (*n* = 110); BNT162b2/ChAdOx1-S (*n* = 114)	S-specific IgG geometric mean titers: At D28 after 2nd dose inoculation, there was a similar titer between the ChAdOx1-S/BNT162b2 and BNT162b2/BNT162b2, but that in ChAdOx1-S/BNT162b2 were significantly higher than that in ChAdOx1-S/ChAdOx1-S and BNT162b2/ChAdOx1-S Neutralization antibody against pseudovirus-wild type-SARS-CoV-2: At D28 after 2nd dose inoculation, there was a similar PVNT_50_ between the ChAdOx1-S/BNT162b2 and BNT162b2/BNT162b2, but that in ChAdOx1-S/BNT162b2 were significantly higher than that in ChAdOx1-S/ChAdOx1-S and BNT162b2/ChAdOx1-S S-specific T cell immune response: At D28 after 2nd dose inoculation, the number of IFN-γ+T cell per 10^6^ PBMC in ChAdOx1-S/BNT162b2 was more than that in ChAdOx1-S/BNT162b2, BNT162b2/BNT162b2 and BNT162b2/ChAdOx1-S Adverse events: Within D28 after 2nd dose inoculation, the incidence of systemic adverse events was increased in heterologous vaccine group as compared to their homologous vaccine group, but no significant difference between those vaccine schedules Within D28 after 2nd dose inoculation, there were four serious adverse events across all groups, but not related to vaccine immunization
**David H. et al., 2021** [42]	Germany	Prospective study	3 weeks: BNT162b2/BNT162b2 10–12 weeks: ChAdOx1-S/ChAdOx1-S, ChAdOx1-S/BNT162b2	Homologous vaccine group: ChAdOx1-S/ChAdOx1-S (*n* = 38, 33- to 59-year-old); BNT162b2/BNT162b2 (*n* = 174, 29- to 43-year-old) Heterologous vaccine group: ChAdOx1-S/BNT162-b2 (*n* = 104, 29- to 51-year-old)	S1-specific and RBD-specific IgG signal-to cutoff- ratio: At D21–28 after 2nd dose inoculation, the ratio of S1-specific IgG in the ChAdOx1-S/BNT162b2 was more than that in all homologous vaccine groups, but no significant difference At D21–28 after 2nd dose inoculation, the ratio of RBD-specific IgG in ChAdOx1-S/BNT162b2 was similar to that in BNT162b2/BNT162b2 and slightly more than that in the ChAdOx1-S/ChAdOx1-S Index of S1-specific IgG avidity: At D21–28 after 2nd dose inoculation, the index of S1-specific IgG avidity in the ChAdOx1-S/BNT162b2 was significantly higher than that in all homologous vaccine groups Neutralization antibody against pseudovirus-variant-SARS-CoV-2 At D21–28 after 2nd dose inoculation, PVNT_50_ against alpha- and beta- SARS-CoV-2 in the ChAdOx1-S/BNT162b2 was significantly higher than that in all homologous vaccine groups S1-specific T cell immune response: At the D21–28 after 2nd inoculation, the production of IFN-γ in the ChAdOx1-S/BNT162b2 was significantly higher than that in in all homologous vaccine groups Adverse events (within 24 h after 2nd dose inoculation): No serious adverse events were reported across all groups The incidence of systemic adverse event in the ChAdOx1-S/BNT162b2 was slightly more than in ChAdOx1-S/ChAdOx1-S and less than that in BNT162b2/BNT162b2 and ChAdOx1-S prime
**Gram M.A. et al.** [40]	Denmark	Clinical study	82 days	Heterologous vaccine group: ChAdOx1-S/BNT162b2 (*n* = 88,050) ChAdOx1-S/mRNA-1273 (*n* = 44,501) Median age of 45 and 46 years at the first and second dose	A reduction in the risk of SARS-CoV-2 infection when combining the ChAdOx1 and an mRNA vaccine. The vaccine effectiveness (VE) against SARS-CoV-2 infection when combining the ChAdOx1 and an mRNA vaccine was 88%. The VE of ChAdOx1/mRNA is similar to the two doses of the BNT162b2 mRNA vaccine. No COVID-19 related hospitalizations were observed after the second dose. No COVID-19 related deaths were observed after neither the first dose ChAdOx1 nor the ChAdOx1/mRNA vaccine schedule.

S, spike protein; RBD, receptor-binding domain; ID_50_, 50% inhibitory dilution; PVNT_50_, 50% of pseudovirus neutralization titer; NR, not reported; S1, S1 domain of spike protein.

## Data Availability

The data presented in this study are available on request from the corresponding author.
